# Diagnosis and management of endometriosis-associated infertility Number 4 – 2026

**DOI:** 10.61622/rbgo/2026FPS4

**Published:** 2026-04-16

**Authors:** Agnaldo Lopes da Silva, Rívia Mara Lamaita, Sérgio Podgaec, Ricardo de Almeida Quintarios, Paula Andrea Navarro, Rui Ferriani

**Affiliations:** Universidade Federal de Minas Gerais Belo Horizonte MG Brazil Universidade Federal de Minas Gerais, Belo Horizonte, MG, Brazil; Universidade Federal de Minas Gerais Belo Horizonte MG Brazil Universidade Federal de Minas Gerais, Belo Horizonte, MG, Brazil; Faculdade de Medicina Universidade de São Paulo São Paulo SP Brazil Faculdade de Medicina, Universidade de São Paulo, São Paulo, SP, Brazil; Faculdade Israelita de Ciências da Saúde Albert Einstein São Paulo SP Brazil Faculdade Israelita de Ciências da Saúde Albert Einstein, São Paulo, SP, Brazil; Faculdade Porto Dias Belém PA Brazil Faculdade Porto Dias, Belém, PA, Brazil; Faculdade de Medicina de Ribeirão Preto Universidade de São Paulo Ribeirão Preto SP Brazil Faculdade de Medicina de Ribeirão Preto, Universidade de São Paulo, Ribeirão Preto, SP, Brazil; Faculdade de Medicina de Ribeirão Preto Universidade de São Paulo Ribeirão Preto SP Brazil Faculdade de Medicina de Ribeirão Preto, Universidade de São Paulo, Ribeirão Preto, SP, Brazil

The National Commissions Specializing in Assisted Reproduction and Endometriosis of the Brazilian Federation of Gynecology and Obstetrics Associations (Febrasgo) endorse this document. The content is based on scientific evidence on the proposed topic, and the results presented contribute to clinical practice.

## Key points

Endometriosis-associated infertility is multifactorial and heterogeneous, and its impact extends across the reproductive continuum, influencing fertility potential, pregnancy course, and obstetric outcomes.
Diagnosis should rely on structured clinical evaluation and expert imaging, with transvaginal ultrasound as the first-line tool and selective use of magnetic resonance imaging.
Routine diagnostic laparoscopy is not indicated in asymptomatic infertile women with normal imaging.
Endometriosis may impair fertility through inflammatory, oxidative, anatomical, ovarian, and endometrial mechanisms, which often act concurrently.
Ovarian endometriomas and ovarian surgery are associated with reduced ovarian reserve.
Hormonal medical treatment does not improve natural fertility outcomes.
Surgery may benefit selected women, but reproductive gain must be balanced against ovarian damage risk.

## Recommendations

Management of endometriosis-associated infertility should be individualized and based on an integrated assessment of female age, duration of infertility, ovarian reserve, disease phenotype, the presence of other infertility-related factors (such as male or tubal factors), symptom burden, prior treatments, and patient reproductive goals.
Diagnosis should rely on a structured clinical and imaging approach, with transvaginal ultrasound as the first line modality, and laparoscopy reserved for cases in which surgical treatment is planned or symptoms persist despite noninvasive evaluation.
Hormonal medical treatment should not be prescribed with the aim of improving fertility outcomes in women seeking pregnancy, as it does not increase conception rates and may delay time to pregnancy.
When surgery is indicated, laparoscopic management should prioritize complete excision of superficial endometriotic lesions, as ablative or purely diagnostic approaches are no longer considered standard.
Surgical excision of ovarian endometriomas should not be routinely performed before assisted reproductive technology and should be reserved for well-defined indications such as significant pain, suspicion of malignancy, rapid cyst growth, or technical barriers to oocyte retrieval.
Assisted reproductive technologies should be prioritized in women with endometriosis-associated infertility who are aged 35 years or older, have prolonged infertility, diminished ovarian reserve, advanced disease, concomitant male factor infertility and/or tubal damage, or who have failed prior surgical treatment. Fertility preservation counseling should be offered to women at increased reproductive risk, ideally before ovarian surgery and at younger ages, to mitigate anticipated loss of ovarian reserve.
The Endometriosis Fertility Index may be used to guide postoperative counseling and timing of referral to assisted reproductive technologies, but clinical decisions should always integrate ovarian reserve markers, reproductive history, and patient preferences.
Women with endometriosis should receive comprehensive, evidence-based counseling not only regarding fertility prospects, but also regarding potential pregnancy-related risks, particularly in severe disease phenotypes and when adenomyosis is present, ensuring an integrated reproductive and obstetric perspective.

### Background

Endometriosis is a chronic inflammatory condition that affects women of reproductive age and is strongly associated with infertility.^(1,2)^ It is associated with a wide spectrum of reproductive dysfunctions, including impaired folliculogenesis, compromised oocyte quality, altered tubal function, reduced endometrial receptivity, and diminished ovarian reserve, resulting from disease-related mechanisms and/or previous ovarian surgery.^(2-4)^ Despite its impact on reproductive outcomes, endometriosis is still frequently underdiagnosed and managed inconsistently, especially in women undergoing infertility evaluation.^(1,2)^

The relationship between endometriosis and infertility is complex and multifactorial. Pelvic inflammation, anatomical distortion, follicular oxidative stress, ovarian endometriomas, and molecular alterations in the eutopic endometrium may coexist and variably affect fecundity, leading to marked interindividual variability in reproductive outcomes.^(3,4)^ Advances in imaging, minimally invasive surgery, and assisted reproductive technologies have changed how endometriosis-associated infertility is approached in clinical practice. At the same time, increasing awareness of the potential harm associated with unnecessary or repeated ovarian surgery has reinforced the importance of cautious and individualized management strategies.^(4,5)^

Current international guidelines, particularly those from the European Society of Human Reproduction and Embryology, emphasize that the management of endometriosis-associated infertility should not be based solely on disease stage. Instead, treatment decisions should be based on an integrated assessment that includes age, duration of infertility, ovarian reserve, disease phenotype, symptom burden, the presence of other infertility-related factors, and previous treatments.^(5)^ Assisted reproductive technologies, especially in vitro fertilization, play a central role in many clinical scenarios, whereas surgery should be reserved for well-defined indications, such as symptom control or technical barriers to assisted reproduction.^(3-5)^Chart 1 compares key recommendations from major international societies regarding the diagnosis and management of endometriosis-associated infertility.

**Chart 1:** Position statements from major societies on endometriosis-associated infertility

	ESHRE	ASRM	FEBRASGO Position Statement
**Diagnostic strategy**	Imaging based diagnosis. Laparoscopy reserved for selected cases	Laparoscopy not routine in asymptomatic infertility	Structured clinical and imaging approach. Surgery only when treatment is planned
**Medical treatment for fertility**	Not recommended to improve fertility	No evidence of fertility benefit	Not indicated for fertility enhancement
**Surgery for superficial disease**	May improve spontaneous pregnancy	Benefit in selected cases	Perform operative treatment when laparoscopy is indicated
**Surgery for ovarian endometrioma**	Not routine before ART	Avoid surgery solely to improve ART outcomes	Selective indication. Prioritize ovarian reserve preservation
**Surgery for deep endometriosis**	Symptom driven indication	Surgery not for fertility alone	Surgery for pain or organ dysfunction. ART prioritized for fertility
**Role of ART**	Central role in unfavorable prognosis	IVF preferred in advanced disease or failed surgery	Early ART when age, reserve, or disease severity are unfavorable
**Fertility preservation**	Recommended in selected cases	Consider before ovarian surgery	Strongly encouraged before ovarian surgery in high-risk women

The objective of this FEBRASGO Position Statement is to critically review and synthesize the best available evidence on the diagnosis and management of endometriosis-associated infertility. By integrating data from systematic reviews, randomized trials, and international guidelines, this document aims to provide practical, evidence-based recommendations that support individualized, patient centered decision making, while minimizing overtreatment and avoiding unjustified delays in fertility-oriented care.

### What is the impact of endometriosis on female fertility?

Endometriosis affects female fertility through several mechanisms, interfering with ovulation, fertilization, embryo development, and implantation. These mechanisms frequently coexist, and their relative contribution varies according to disease phenotype and the individual reproductive context.^(2-4,6)^

Anatomical distortion plays a relevant role in moderate and severe disease. Chronic inflammation promotes fibrosis and adhesion formation, leading to impaired tubal motility, altered oocyte capture, and reduced gamete transport. Ovarian endometriomas may further disrupt tubo-ovarian anatomy and contribute to mechanical infertility, particularly in advanced stages of disease.^(3,6)^

In minimal and mild endometriosis, infertility is predominantly mediated by a proinflammatory peritoneal environment rather than by mechanical factors. Increased concentrations of activated macrophages, prostaglandins, and inflammatory cytokines negatively affect sperm function, fertilization, and early embryo development, even in the absence of overt anatomical distortion.^(3,7)^ In addition, oxidative stress associated with alterations in the peritoneal, serum, and/or follicular microenvironments may further impair oocyte quality.^(8)^

Ovarian function is frequently compromised, especially in women with ovarian endometriomas. The endometrioma microenvironment is characterized by oxidative stress, iron overload, and mitochondrial dysfunction, which damage granulosa cells and oocytes, resulting in reduced oocyte competence and impaired embryo development. Clinically, these mechanisms are reflected by lower antimüllerian hormone (AMH) levels and reduced antral follicle counts, indicating diminished ovarian reserve.^(7,9,10)^

Surgical treatment of endometriomas may further aggravate ovarian damage. Systematic reviews and meta-analyses consistently demonstrate a significant postoperative decline in AMH levels, with greater impact in bilateral disease and after repeat surgery. These findings highlight the iatrogenic risk associated with ovarian surgery and reinforce the need for cautious, individualized decision making when fertility preservation is a priority.^(11)^

Endometrial receptivity may also be altered in women with endometriosis due to progesterone resistance and dysregulated estrogen signaling. Molecular alterations in the eutopic endometrium impair decidualization and embryo endometrial interaction, further contributing to reduced implantation potential.^(12)^

Evidence from oocyte donation models supports the central role of oocyte related factors in endometriosis-associated infertility. Lower implantation and pregnancy rates are observed when oocytes from women with endometriosis are transferred to disease free recipients, whereas recipients with endometriosis receiving oocytes from healthy donors achieve reproductive outcomes comparable to controls.^(6)^

Endometriosis interferes with female fertility through intertwined anatomical, inflammatory, and molecular mechanisms that affect multiple stages of the reproductive process. The relative contribution of each mechanism varies across individuals, underscoring the need for personalized management strategies that integrate expectant management, surgery, and assisted reproductive technologies according to clinical context and reproductive goals.^(3,6,12)^

### How should endometriosis be diagnosed in women undergoing infertility evaluation?

In women undergoing infertility evaluation, endometriosis should be suspected when infertility is associated with pelvic pain symptoms or when no alternative cause of infertility is identified after standard assessment. Current evidence supports a diagnostic approach that prioritizes clinical assessment and imaging, reserving surgical confirmation for selected situations.^(2-5,13)^

Clinical suspicion should be raised in infertile women reporting dysmenorrhea that is progressive or poorly responsive to analgesics, deep dyspareunia, cyclic bowel or urinary symptoms, or chronic pelvic pain. While these symptoms are nonspecific when considered individually, their association with infertility increases the likelihood of underlying endometriosis. Physical examination may reveal uterosacral tenderness, nodularity, reduced uterine mobility, or adnexal masses suggestive of endometrioma. However, a normal examination does not exclude disease, particularly in minimal or superficial forms, and should not delay further investigation when clinical suspicion exists.^(2-4,13)^

Transvaginal ultrasound is recommended as the first line imaging modality in infertile women with suspected endometriosis. It provides high diagnostic accuracy for ovarian endometriomas and, when performed systematically by trained operators, allows reliable identification of deep endometriosis involving the posterior compartment, bowel, or bladder. The use of standardized scanning protocols improves detection of deep disease and supports treatment planning. A normal ultrasound does not exclude superficial peritoneal endometriosis, and imaging findings should always be interpreted in conjunction with clinical presentation.^(4,5)^

Magnetic resonance imaging should be used as a complementary modality when ultrasound is inconclusive, when deep or extrapelvic endometriosis is suspected, or when detailed preoperative anatomical assessment is necessary. MRI offers broader anatomical assessment and is particularly useful for evaluating bowel, bladder, ureteral, or diaphragmatic involvement, although its sensitivity for superficial disease remains limited.^(5)^

The role of diagnostic laparoscopy in infertility evaluation has been substantially redefined. Current ASRM and ESHRE guidelines do not recommend routine diagnostic laparoscopy in asymptomatic infertile women with normal imaging, as detection of minimal or mild disease has a limited impact on reproductive outcomes and does not justify surgical risk or treatment delay. Laparoscopy should be reserved for women with persistent symptoms, imaging confirmed disease requiring surgical management, or when histological confirmation is expected to influence individualized treatment decisions. When performed, laparoscopy should be therapeutic rather than purely diagnostic.^(2,3,5,13)^

At present, no noninvasive biomarker has sufficient accuracy to replace imaging or surgery in the diagnosis of endometriosis. Serum CA 125 lacks sensitivity for early disease and shows significant overlap with other benign or inflammatory conditions, limiting its utility in infertility evaluation. Emerging biomarkers remain investigational and are not recommended for routine clinical use.^(2,4)^

Diagnosis of endometriosis in infertile women should follow a stepwise, integrated strategy based on clinical suspicion, systematic imaging, and selective use of laparoscopy. This approach minimizes unnecessary surgery, reduces diagnostic delay, and allows timely alignment of treatment decisions with reproductive goals, disease severity, and patient preferences.

### How should women with suspected or confirmed endometriosis be initially evaluated in the context of infertility?

The initial evaluation of women with suspected or confirmed endometriosis presenting with infertility should follow a structured, couple centered approach that integrates reproductive history, assessment of ovarian reserve, evaluation of pelvic anatomy, and identification of coexisting female and male factors that may independently or synergistically impair fertility.^(3,4,14,15)^ Current guidelines emphasize that endometriosis-associated infertility is multifactorial and that isolated assessment of disease presence or stage is insufficient for prognostic counseling or treatment planning.^(3,14)^

A detailed reproductive history represents the cornerstone of initial evaluation and should include female age, duration of infertility, prior pregnancies, and previous fertility treatments.^(14,15)^ Female age is a dominant prognostic determinant, as advancing age and endometriosis exert additive negative effects on fecundity, ovarian reserve, and reproductive outcomes.^(4,14)^ The duration of infertility and a history of prior pregnancy provide additional prognostic information and are incorporated into validated predictive models, including the Endometriosis Fertility Index.^(16)^

Assessment of ovarian reserve is a critical component of the initial workup. Measurement of AMH and/or antral follicle count is recommended for all women with endometriosis seeking pregnancy, given the consistent association between endometriosis, particularly ovarian endometriomas, and diminished ovarian reserve.^(3,14)^ Evidence from population studies and meta-analyses demonstrates that ovarian reserve may be reduced even in the absence of prior ovarian surgery, underscoring the importance of early evaluation and counseling. Ovarian reserve markers should be interpreted in the context of age, disease phenotype, and surgical history, and should guide discussions regarding timing of treatment and fertility preservation strategies when appropriate.^(4,14)^

Assessment of pelvic anatomy and tubal patency is essential, as endometriosis may compromise fertility through tubal obstruction, peritubal adhesions, and fimbrial dysfunction.^(3,15)^ Hysterosalpingography or hysterosalpingo contrast sonography are appropriate first line tests for tubal assessment. Laparoscopy should be reserved for women with persistent symptoms, imaging confirmed disease, or when surgical treatment is planned. Routine diagnostic laparoscopy in asymptomatic infertile women with normal imaging is not recommended by major societies.^(14,15)^

Systematic uterine evaluation should also be performed, with particular attention to the frequent coexistence of adenomyosis, which may independently impair implantation and reproductive outcomes.^(3)^ Transvaginal ultrasound is the first line modality, with pelvic magnetic resonance imaging reserved for equivocal cases or when detailed anatomical assessment is required.^(15)^Parallel evaluation of the male partner is mandatory in all couples presenting with endometriosis-associated infertility, as male factor infertility accounts for infertility in a substantial proportion of cases and may significantly influence treatment selection and reproductive outcomes.^(15)^ Semen analysis should be obtained early in the evaluation to avoid unnecessary delays or inappropriate female focused interventions.

When surgical findings are available, disease staging and prognostic assessment should be integrated into clinical decision making. While the revised American Society for Reproductive Medicine classification provides anatomical staging, its correlation with fertility outcomes is limited.^(3)^ The Endometriosis Fertility Index is currently the most extensively validated tool for predicting the likelihood of non-assisted conception after surgery and may guide postoperative counseling and timing for referral to assisted reproductive technologies.^(16)^

Initial evaluation should synthesize clinical, laboratory, imaging, and, when available, surgical information to support individualized counseling and timely treatment planning. This integrated approach allows clinicians to balance the potential for spontaneous conception against the time sensitive nature of fertility, particularly in women with advancing age or compromised ovarian reserve.^(4,14)^

### Does medical treatment improve fertility outcomes in women with endometriosis-associated infertility?

Available evidence shows that medical treatment, when used alone, does not improve fertility outcomes.^(2,3,5,17)^ Although hormonal therapies are effective for pain control, their mechanism of action is based on suppression of ovulation and estrogen production, which inherently precludes conception during treatment and may delay time to pregnancy.^(2,3)^

This position is strongly supported by international guidelines. The American Society for Reproductive Medicine states that there is no evidence that medical treatment of endometriosis improves fertility, and randomized controlled trials have shown no difference in pregnancy rates between hormonal suppression and expectant management in women attempting conception.^(3)^ These conclusions are consistently endorsed by scientific reviews and expert consensus documents.^(2,17)^

High quality evidence from systematic reviews further reinforces this recommendation. A landmark Cochrane review evaluating ovulation suppression for endometriosis-associated infertility demonstrated no benefit of hormonal therapy compared with placebo or no treatment for achieving pregnancy, regardless of the agent used, including danazol, progestins, combined oral contraceptives, or GnRH agonists.^(18)^ Subsequent overviews of Cochrane reviews have confirmed the lack of efficacy of medical therapy for fertility enhancement, in contrast to the benefit observed with surgical treatment in selected patients.^(17)^

The role of medical therapy becomes more complex in the setting of assisted reproductive technologies. Prolonged GnRH agonist pretreatment before IVF has been explored as a strategy to improve outcomes, largely driven by early studies reporting higher clinical pregnancy rates.^(3)^ However, more recent evidence, including updated Cochrane analyses and randomized controlled trials, has raised uncertainty regarding its impact on live birth rates, with the overall quality of evidence rated as low or very low.^(19,20)^ Consequently, routine hormonal pretreatment before IVF is not universally recommended and should be considered selectively, particularly in women with advanced disease or previous ART failure.^(2,19)^

Postoperative hormonal suppression represents another area of clinical debate. Systematic reviews and meta-analyses show that overall pregnancy and live birth rates are not significantly improved by postoperative hormonal therapy in women wishing to conceive.^(9,10)^ While subgroup analyses suggest a modest benefit when GnRH agonists are used for at least three months, this potential advantage must be carefully weighed against the loss of reproductive time, especially in women of advanced reproductive age or with diminished ovarian reserve.^(21)^

Importantly, medical therapy may delay attempts at conception, a relevant concern in women with time-sensitive fertility. Because hormonal treatments suppress ovulation, such delays may negatively affect cumulative pregnancy rates.^(2,15)^ For this reason, guidelines consistently emphasize that fertility oriented management in endometriosis should prioritize strategies with demonstrated reproductive benefit, including surgical treatment in selected cases, ovulation induction with intrauterine insemination for minimal or mild disease, and in vitro fertilization as the most effective option for many patients.^(2,3,15)^

Overall, the available evidence supports the conclusion that medical treatment should not be used as primary therapy to improve fertility in women with endometriosis. Hormonal suppression plays an important role in pain management and may have a limited, individualized role as adjunctive therapy in ART protocols, but it does not enhance natural fecundity and may delay conception when pregnancy is the primary goal.^(2,3,17)^

### What is the role of surgery in the management of endometriosis-associated infertility?

Surgery has a selective role in the management of endometriosis-associated infertility and should be considered on an individual basis.^(2-4)^ Surgical indication should be guided by disease phenotype, symptom burden, ovarian reserve status, patient age, and reproductive goals, rather than by infertility alone.^(3,4,22)^

The strongest evidence supporting surgical intervention concerns minimal and mild peritoneal endometriosis. Randomized controlled trials and Cochrane meta-analyses demonstrate that laparoscopic excision or ablation of superficial lesions significantly increases spontaneous pregnancy rates compared with diagnostic laparoscopy alone.^(22)^ Accordingly, when laparoscopy is performed in infertile women with suspected endometriosis, operative treatment should be undertaken rather than diagnostic exploration alone.^(3,22)^

In moderate and severe disease, evidence derives mainly from observational studies. Conservative surgery has been associated with improved spontaneous conception rates compared with expectant management, particularly in women without additional infertility factors.^(3)^ However, fertility outcomes following surgery are heterogeneous, and the absence of randomized trials reinforces the need for careful patient selection.^(2,3)^

Deep infiltrating endometriosis represents a particularly complex scenario. Observational data suggest that surgery may improve spontaneous pregnancy rates in symptomatic women.^(23)^However, comparative studies and meta-analyses indicate that first line surgery and first line assisted reproductive technology achieve similar pregnancy and live birth rates in infertile women with deep endometriosis.^(24)^ In asymptomatic patients whose primary goal is pregnancy, ART may therefore be preferred, whereas surgery should be reserved for women with significant pain or organ dysfunction.^(4,23)^

The management of ovarian endometriomas remains the most controversial aspect of surgical decision making. Robust evidence demonstrates that endometrioma surgery, particularly cystectomy, is associated with a significant and sustained reduction in ovarian reserve, reflected by declines in AMH levels and antral follicle count.^(24,25)^ Importantly, multiple systematic reviews show that surgical removal of endometriomas does not improve IVF outcomes and may reduce oocyte yield without increasing live birth rates.^(25)^Alternatively, surgical removal of endometriomas may improve the chances of natural conception and can be considered in younger women with preserved ovarian reserve, in the absence of associated tubal or male factor infertility, while taking into account patient preferences and access to care.

Accordingly, international guidelines recommend that asymptomatic endometriomas should not be routinely removed prior to IVF, as surgery offers no reproductive benefit and may be harmful.^(3)^ Surgical intervention may be considered in selected cases, such as large cysts compromising follicle access, suspicion of malignancy, or severe pain, with thorough counseling regarding the potential impact on ovarian reserve.^(3,25)^

Repeat surgery should be approached with caution, as cumulative ovarian damage is well documented. Evidence consistently shows that repeat operative intervention rarely improves fertility and is associated with cumulative ovarian damage.^(3)^ When initial surgery fails to restore fertility, assisted reproductive technology is generally more effective than additional surgical treatment.^(3)^

Surgery is an important but non universal strategy in endometriosis-associated infertility. Laparoscopic treatment of superficial peritoneal disease improves spontaneous pregnancy rates and should be performed when laparoscopy is indicated.^(22)^ In contrast, ovarian endometriomas and deep endometriosis require a conservative, individualized approach, with IVF prioritized when fertility is the primary objective and symptoms are minimal.^(3,4,25)^ Ongoing randomized trials, including the SVIDOE study, are expected to further refine the role of surgery versus ART in this setting.^(26)^

### How does disease stage, symptom burden, and ovarian reserve influence treatment selection?

Disease stage is an important determinant of treatment selection in women with endometriosis-associated infertility, as it influences both prognosis and the likelihood of spontaneous conception. In minimal or mild disease (stage I/II), fertility impairment is predominantly related to inflammatory and functional mechanisms rather than anatomical distortion. In this context, expectant management or conservative strategies are often appropriate, as available evidence does not support a consistent benefit of surgical treatment of superficial peritoneal endometriosis on spontaneous pregnancy rates.^(2,3,15)^Ovulation induction with intrauterine insemination may also be considered in carefully selected women with preserved ovarian reserve and no additional infertility factors.^(3)^

In contrast, moderate to severe disease (stage III/IV), characterized by ovarian endometriomas, deep infiltrating endometriosis, or extensive adhesions, is associated with a substantially lower probability of spontaneous conception. In these patients, anatomical distortion, tubal dysfunction, and compromised ovarian reserve frequently justify expedited referral to assisted reproductive technology or a combined surgical and ART approach when clinically indicated.^(3,4,15)^ Disease stage therefore plays a central role in prognostic counseling and in defining the intensity and timing of intervention.

Symptom burden is a key modifier of treatment prioritization. Although pain severity correlates poorly with disease stage, symptoms such as dysmenorrhea, chronic pelvic pain, and dyspareunia strongly influence patient-centered decision-making. In women whose dominant complaint is pain, medical therapy or surgery may be prioritized even when fertility is a concurrent goal.^(2,5)^Conversely, asymptomatic infertile women may reasonably proceed directly to ART, reserving surgery for refractory pain or clearly defined anatomical barriers to conception.^(2,3)^

Functional impact and quality of life further refine individualized management. When disease-related symptoms significantly interfere with daily activities, sexual health, or psychosocial well-being, a more comprehensive approach may be justified, including surgical treatment and multidisciplinary care.^(2,5)^ Patient-reported outcomes therefore complement anatomical staging and should inform treatment selection.

Assessment of ovarian reserve is central to reproductive decision-making. Measurement of AMH and antral follicle count provides critical prognostic information, particularly in women with ovarian endometriomas or prior ovarian surgery.^(4,15)^ Diminished ovarian reserve favors earlier use of ART and argues against unnecessary delays related to prolonged expectant management or repeated surgical intervention.

Integrating disease stage, symptom burden, functional impact, and ovarian reserve enable truly individualized care. A young woman with stage I/II disease, preserved ovarian reserve, and minimal symptoms may be managed conservatively, whereas an older woman with stage III/IV disease, significant symptoms, and reduced ovarian reserve is more likely to benefit from early ART, with surgery reserved for symptom control or specific anatomical indications.^(2-4,15)^ This integrated approach reflects current international consensus and aims to maximize reproductive outcomes while minimizing morbidity and loss of reproductive potential.

### How should ovarian endometriomas be managed in women seeking pregnancy?

Ovarian endometriomas are present in up to 50 percent of women with endometriosis-associated infertility and are consistently associated with reduced ovarian reserve and impaired reproductive potential, reflected by lower AMH levels and antral follicle counts.^(4,27,28)^ Diagnosis is usually established by transvaginal ultrasound, which provides high diagnostic accuracy and enables longitudinal assessment of cyst size and ovarian morphology.^(27,29)^Management decisions should integrate infertility status, symptom burden, ovarian reserve, cyst characteristics, and reproductive plans.

Expectant management is appropriate for asymptomatic women seeking pregnancy, particularly when ovarian reserve is already reduced, the endometrioma is small, and follicle access during assisted reproductive technology is not compromised.^(15,27,29)^ This strategy avoids iatrogenic ovarian damage, preserves existing ovarian reserve, and prevents unnecessary delays in fertility treatment. International guidelines strongly support observation in the absence of pain, suspicious imaging features, or technical barriers to ART.^(3,27,29)^

Surgical intervention should be reserved for clearly defined indications, including significant pain, large cysts, usually greater than 4 cm, suspicion of malignancy, rapid cyst growth, or technical impediments to oocyte retrieval.^(27)^ Available surgical approaches include cystectomy, ablative techniques, or combined methods. While cystectomy offers lower recurrence rates and superior pain control, it is associated with a greater risk of postoperative ovarian reserve loss compared with ablative techniques.^(28,30)^Ablative approaches may better preserve ovarian tissue but are associated with higher recurrence rates. The choice of technique should be individualized, performed by experienced surgeons, and preceded by detailed counseling regarding potential reproductive impact.^(27,30)^

Both the presence of an endometrioma and its surgical removal may adversely affect ovarian reserve. Endometriomas can impair ovarian function through chronic inflammation and local tissue damage, while surgery, particularly cystectomy, may further reduce ovarian reserve, especially in bilateral disease or repeat procedures.^(25,27,28)^Preoperative assessment of ovarian reserve is therefore essential, and fertility preservation strategies should be discussed in women at risk of further reserve loss.

Current evidence consistently shows that routine surgical excision of endometriomas before IVF or ICSI does not improve reproductive outcomes. Meta analyses and systematic reviews demonstrate comparable live birth and clinical pregnancy rates with or without prior surgery, while surgery is associated with lower AMH levels and reduced oocyte yield.^(25,30)^ Accordingly, surgery should not be performed solely to enhance ART outcomes and should be reserved for selected clinical indications.

Management of ovarian endometriomas in women seeking pregnancy should follow an individualized, multidisciplinary approach. Expectant management is preferred for asymptomatic women with preserved access to ART, whereas surgery is reserved for pain, suspicion of malignancy, or technical impediments. When surgery is indicated, ovarian sparing techniques and fertility preservation counseling are essential to minimize reproductive harm.^(5,27-30)^

### What is the role of surgery for deep endometriosis in infertile women?

Surgery for deep infiltrating endometriosis in infertile women should be primarily indicated for symptom control and preservation of organ function, rather than for isolated improvement of fertility outcomes. International guidelines from ESHRE, ACOG, and the World Endometriosis Society consistently recommend restricting surgical intervention to women with severe pain, bowel, ureteral or bladder involvement, progressive organ dysfunction, or significant anatomical distortion that precludes access to assisted reproductive technology.^(2,3,31)^

In symptomatic women who desire pregnancy, surgical excision of deep endometriosis may result in spontaneous conception in a subset of carefully selected patients. Observational studies and systematic reviews suggest that restoration of pelvic anatomy and reduction of inflammatory burden can improve fertility potential in women with severe pain or obstructive disease.^(32)^ However, robust evidence supporting surgery as a fertility enhancing intervention in asymptomatic infertile women is lacking, and comparative studies indicate that reproductive outcomes after first line surgery or first line assisted reproduction are broadly similar in this population.^(32,33)^

In asymptomatic women whose primary goal is pregnancy, surgery should not be routinely performed solely to improve fertility. Surgical treatment of deep endometriosis is associated with a non-negligible risk of major complications, postoperative adhesions, and potential adverse effects on ovarian reserve or pelvic function, which may outweigh uncertain reproductive benefits.^(2,33)^ In these cases, direct referral to assisted reproductive technology is generally favored, particularly in women of advanced reproductive age, those with diminished ovarian reserve, or concomitant male factor infertility.

Management of deep endometriosis requires a multidisciplinary approach in specialized tertiary centers, given the technical complexity of surgery and the frequent involvement of bowel, urinary tract, or other pelvic organs. Collaboration with colorectal, urological, and, when necessary, thoracic surgeons is essential to minimize morbidity and optimize outcomes.^(4,31)^ The primary objectives of surgery are pain relief, preservation of organ function, and improvement in quality of life. Fertility considerations should be addressed through shared decision making, clearly distinguishing symptom driven indications for surgery from fertility-oriented treatment goals.^(33,34)^

Surgery for deep infiltrating endometriosis in infertile women should be symptom driven rather than fertility driven. While surgical excision may facilitate spontaneous conception in selected symptomatic patients, assisted reproductive technology remains the preferred first line fertility treatment for asymptomatic women. Ongoing randomized trials comparing surgery with assisted reproduction will further refine treatment sequencing, but current evidence supports an individualized, patient centered approach that prioritizes safety, symptom control, and reproductive timing.

### When should assisted reproductive technologies be prioritized in endometriosis-associated infertility?

Endometriosis-associated infertility is multifactorial, involving inflammatory, tubal, ovarian, and endometrial mechanisms that ultimately reduce fecundity .^(2,15,35)^ Assisted reproductive technologies should be considered early in women with endometriosis-associated infertility who present adverse prognostic factors. These include female age of 35 years or older, infertility duration longer than two years, diminished ovarian reserve, advanced disease (stage III or IV), associated male factor infertility, tubal damage, failed prior surgical treatment, or significant pelvic anatomical distortion.^(2,15,35)^ In these clinical contexts, ART represents the most effective strategy to achieve pregnancy and should not be unnecessarily delayed by expectant management or non-beneficial interventions. Chart 2 outlines the main clinical scenarios in which assisted reproductive technologies should be prioritized in women with endometriosis-associated infertility.

**Chart 2:** When to prioritize assisted reproductive technology in endometriosis

Prognostic factor	Clinical implication	Suggested approach
Age 35 years or older	Reduced fecundity and oocyte quality	Early referral to ART
Infertility longer than 2 years	Lower probability of spontaneous conception	Avoid prolonged expectant management
Low AMH or reduced AFC	Limited reproductive time window	Prioritize ART
Stage III or IV disease	Anatomical distortion and reduced reserve	ART preferred over expectant management
Prior failed surgery	Low likelihood of benefit from repeat surgery	ART rather than reoperation
Deep endometriosis without pain	Surgery unlikely to improve fertility	ART as first line strategy
Male factor infertility	Reduced chance of spontaneous conception	ART indicated
Tubal damage (bilateral or severe unilateral)	Impaired gamete transport and fertilization	ART preferred

**Figure 1:**
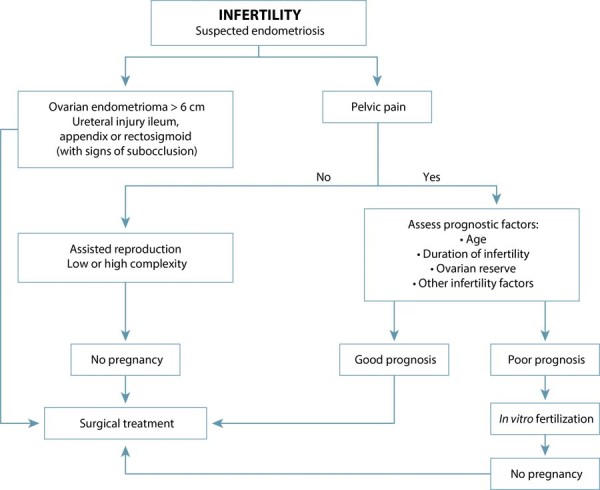
Therapeutic flowchart for patients with clinical suspicion of endometriosis-associated infertility

Figure 1 presents a FEBRASGO-based therapeutic flowchart integrating clinical presentation, prognostic factors, and anatomical findings to guide fertility-oriented management.^(36)^

Female age is a major determinant of treatment selection. After 35 years of age, fecundity declines rapidly, and the negative effects of endometriosis and reproductive aging appear to be additive, with reduced cumulative live birth rates and increased miscarriage risk.^(3,15)^ Similarly, prolonged infertility duration is associated with a progressively lower probability of spontaneous conception, supporting early referral to ART when infertility exceeds two years.^(3,35)^

Assessment of ovarian reserve plays a central role in decision-making. Women with low AMH levels or reduced antral follicle count should be counseled toward early ART in order to maximize reproductive potential before further decline occurs.^(35)^ Advanced endometriosis, prior failed surgery, or extensive pelvic adhesions further justify prioritizing ART, as spontaneous pregnancy rates in these scenarios are low and surgical intervention may carry additional risks without clear fertility benefit.^(3,15,35)^

Surgical treatment before ART should be selective rather than routine. Surgery may be considered in women with severe pain, large endometriomas that impair follicle access, or significant anatomical barriers to oocyte retrieval. However, both endometriosis itself and surgical treatment, particularly ovarian surgery, are associated with further reduction in ovarian reserve, and routine pre-ART surgery does not improve live birth rates.^(2,3)^ Consequently, surgery should not be performed solely to enhance fertility outcomes before ART.

ART outcomes in women with minimal or mild endometriosis are generally comparable to those observed in other infertility etiologies. In contrast, women with advanced disease tend to have lower oocyte yield and reduced live birth rates, reinforcing the importance of individualized counseling and early ART referral in this subgroup.^(3,35)^ In selected cases, a combined approach involving symptom-driven surgery followed by ART may be appropriate, particularly when pain and infertility coexist or when pelvic anatomy limits ART feasibility.^(35)^

ART should be prioritized in women with endometriosis-associated infertility who are older, have prolonged infertility, diminished ovarian reserve, male and/or tubal factor, advanced disease, or failed prior surgery. Treatment decisions must be individualized, integrating age, ovarian reserve, disease severity, prior interventions, and patient preferences. Current guidance from the American Society for Reproductive Medicine and other major societies supports early use of ART in women with unfavorable reproductive prognosis, rather than delaying treatment with ineffective or potentially harmful strategy.^(2,3,15,35)^

### How can prognostic tools, such as the Endometriosis Fertility Index, support clinical decision-making?

The Endometriosis Fertility Index is a validated prognostic tool designed to estimate the probability of spontaneous, non-ART pregnancy after surgical treatment of endometriosis. It should be used as a complementary aid to individualized clinical decision-making rather than as a deterministic predictor of reproductive outcome.^(16,37,38)^ The EFI integrates historical variables, including female age, duration of infertility, and prior pregnancy, with intraoperative findings, most notably the least function score, which reflects residual tubal and ovarian function at the conclusion of surgery. The final score ranges from 0 to 10, with higher values indicating a more favorable reproductive prognosis.^(16,37,38)^

The EFI has been externally validated across multiple populations and consistently demonstrates a strong association with time to spontaneous conception following surgery. A large systematic review and meta-analysis including nearly 4,600 women reported cumulative non-ART pregnancy rates at 36 months ranging from approximately 10 percent for EFI scores of 0–2 to nearly 70 percent for scores of 9–10, with an odds ratio of approximately 1.3 per point increase and moderate overall discriminatory performance.^(16)^Across studies, each one-point increase in EFI score is associated with a clinically meaningful increase in the probability of pregnancy, supporting its role in postoperative fertility counseling.^(38,39)^ Among all EFI components, the least function score consistently emerges as the strongest predictor of fertility outcome, underscoring the importance of preserved tubo-ovarian function.^(16,38,39)^

In clinical practice, the EFI assists in stratifying women into prognostic groups and guiding the timing and intensity of fertility treatment after surgery. Women with high EFI scores, generally 7 or higher, have a favorable likelihood of spontaneous conception and may reasonably attempt natural pregnancy for up to 12 to 24 months before referral to assisted reproductive technologies. In contrast, women with low EFI scores, typically 4 or lower, have a limited probability of non-ART pregnancy and should be counseled toward early referral to ART, avoiding unnecessary delays that may further compromise reproductive outcomes.^(40,41)^ Prospective studies and cost-effectiveness analyses suggest that EFI-guided management improves allocation of ART resources, with the relative benefit of ART increasing as EFI scores decline.^(40,41)^

Although originally conceived as a postoperative score, the EFI may be estimated preoperatively using clinical history and imaging; however, no fully validated non-surgical version currently exists. This expands its utility in shared decision-making before surgery and supports discussions regarding the relative roles of surgery, expectant management, and ART in women with endometriosis-associated infertility.^(37)^

Despite its strengths, the EFI has important limitations. Its predictive accuracy is moderate, and a substantial proportion of fertility outcome variability is explained by factors not captured in the score.^(16,38)^ Some subjectivity exists, particularly in the assessment of the least function score, and ovarian reserve markers such as AMH and antral follicle count are not incorporated, even though they independently influence prognosis.^(16,42)^ In addition, the EFI is more reliable for predicting spontaneous conception than ART outcomes and should not be used in isolation to guide complex fertility decisions.^(35,42)^

The EFI outperforms rASRM staging alone in predicting fertility outcomes, but its greatest value lies in its integration with other key clinical variables, including age, ovarian reserve, duration of infertility, symptom burden, and patient preferences.^(35,38)^When interpreted within this broader clinical context, the EFI represents a valuable tool to support individualized, patient-centered fertility management in women with endometriosis.

### When should fertility preservation be discussed in women with endometriosis?

Fertility preservation should be discussed with selected women with endometriosis who are at increased risk of future loss of reproductive potential, particularly before planned ovarian surgery, in the presence of bilateral or recurrent endometriomas, in women with diminished ovarian reserve, and in those diagnosed at a young age who may defer childbearing.^(43-46)^ This strategy is supported by consistent evidence demonstrating that both endometriosis itself and its surgical treatment, especially ovarian endometrioma excision, are associated with a clinically relevant and sometimes irreversible reduction in ovarian reserve, reflected by declines in AMH levels and antral follicle count.^(44-46)^

International guidelines and expert consensus recommend that counseling regarding fertility preservation should ideally occur before any ovarian surgery, particularly when cystectomy is planned or repeat surgery is anticipated.^(43-46)^Among available techniques, oocyte cryopreservation is the most established and widely recommended option, as it does not require a partner and offers greater reproductive autonomy. Embryo cryopreservation may be considered in women with a stable partner, while ovarian tissue cryopreservation remains experimental in the context of endometriosis and should be reserved for highly selected cases.^(43,46)^

Fertility preservation may be particularly relevant in women with severe disease phenotypes, including large endometriomas, bilateral ovarian involvement, deep infiltrating endometriosis requiring complex surgery, or a history of prior ovarian procedures, all of which are associated with cumulative ovarian damage.^(44-47)^ Women with additional risk factors for premature ovarian insufficiency or evidence of rapidly declining ovarian reserve may also benefit from individualized counseling.^(47)^

Timing is a critical determinant of effectiveness. Evidence consistently shows that fertility preservation yields better outcomes when performed at younger ages, preferably before 35 years, and prior to ovarian surgery, as both oocyte quantity and quality decline with age and are further compromised by surgical ovarian injury.^(45,46,48)^ Nevertheless, fertility preservation is not universally indicated for all women with endometriosis and should not be offered indiscriminately. Instead, it should be individualized based on age, ovarian reserve, disease severity, surgical history, reproductive plans, and patient preferences.^(48)^

Fertility preservation represents a proactive strategy within the broader management of endometriosis-associated infertility, aiming to mitigate anticipated loss of ovarian reserve rather than to treat established infertility. Its role is complementary to surgical and assisted reproductive approaches and should be incorporated into shared decision-making for women at highest reproductive risk.

### How does endometriosis affect reproductive outcomes and pregnancy related complications?

Endometriosis is associated with a clinically meaningful reduction in reproductive potential across the reproductive continuum. Women with endometriosis present lower rates of spontaneous conception and live birth and a higher likelihood of requiring assisted reproductive technologies to achieve pregnancy. Available evidence suggests that live birth rates may be reduced by approximately 10 to 20 percent compared with women without endometriosis, reflecting impaired oocyte competence, altered fertilization, reduced implantation, and dysregulated endometrial function.^(5,49,50)^

Patients should not be advised to become pregnant solely for the purpose of treating endometriosis, as pregnancy does not always lead to improvement in symptoms or a reduction in disease progression.^(5)^

Endometriosis lesions can change in appearance during pregnancy. If an atypical endometrioma is observed on ultrasound, it is recommended to refer the patient to a center with appropriate expertise for evaluation.^(5)^

Pregnancy loss is also more frequent in women with endometriosis. According to large cohort studies and systematic reviews, the risk of first trimester miscarriage is increased by approximately 30 to 70 percent compared with women without the disease. The ESHRE guideline recognizes this association and recommends that clinicians should be aware of the increased risk of miscarriage and ectopic pregnancy in women with endometriosis.^(5,49,50)^

Beyond early pregnancy, endometriosis has been consistently associated with an increased risk of adverse obstetric outcomes. Women with endometriosis have a 20 to 70 percent higher risk of preterm birth and a substantially increased risk of placenta previa. Other complications, including hypertensive disorders of pregnancy, gestational diabetes, malpresentation, and cesarean delivery, also occur more frequently, although the absolute risk increase remains moderate.^(5,49,51)^

Adverse neonatal outcomes are likewise more common. Pregnancies affected by endometriosis are associated with higher rates of low birth weight, small for gestational age neonates, admission to neonatal intensive care units, and perinatal mortality. Importantly, these associations are observed in both spontaneous and assisted conceptions, suggesting that endometriosis itself contributes to obstetric and neonatal risk, independent of the mode of conception.^(5,50,51)^

Disease phenotype influences the magnitude of risk. Women with severe disease, deep infiltrating endometriosis, or concomitant adenomyosis consistently present the highest rates of adverse reproductive and obstetric outcomes. These findings are biologically plausible and are thought to be related to chronic inflammation, progesterone resistance, impaired decidualization, and abnormal placentation.^(4,5,49)^

Current evidence supports the recognition of endometriosis as a condition associated with increased reproductive and obstetric risk. In line with the ESHRE guideline, women with endometriosis should receive appropriate counseling regarding potential pregnancy related risks. While routine intensification of antenatal surveillance is not universally recommended, individualized obstetric follow up may be considered, particularly in women with severe disease phenotypes or additional risk factors.^(5,49)^

### What are the main risks of overtreatment or delayed treatment in endometriosis-associated infertility?

The management of endometriosis-associated infertility requires careful avoidance of two opposing but equally harmful strategies: overtreatment and unjustified treatment delay. Chart 3 summarizes key practical recommendations and common pitfalls in the fertility-oriented management of women with endometriosis. These recommendations reflect current international consensus and underpin the principles outlined in this Position Statement.

**Chart 3:** Do’s and Don’ts in endometriosis-associated infertility

DO	DON’T
Base clinical decision-making on an integrated assessment of age, ovarian reserve, disease phenotype, symptom burden, infertility duration, concomitant male factor infertility and/or tubal damage and reproductive goals.	Do not rely exclusively on disease stage to determine fertility prognosis or therapeutic strategy.
Use transvaginal ultrasound as the first-line diagnostic tool, complemented by magnetic resonance imaging when deep or complex disease is suspected.	Do not perform diagnostic laparoscopy routinely in asymptomatic infertile women with normal imaging findings.
Incorporate ovarian reserve markers early in counseling to guide treatment sequencing and timing of assisted reproductive technologies.	Do not prescribe hormonal suppression with the expectation of improving natural fertility or as a substitute for effective fertility-oriented treatment.
Prioritize assisted reproductive technologies in women with unfavorable prognostic factors, including advanced age, prolonged infertility, diminished ovarian reserve, or advanced disease.	Do not remove ovarian endometriomas systematically before ART in the absence of pain, malignancy suspicion, or technical impairment of follicle access.
Reserve surgical intervention for well-defined indications such as pain refractory to medical management, organ dysfunction, suspicion of malignancy, or technical barriers to ART.	Do not delay referral to assisted reproduction in women with advancing age, reduced ovarian reserve, or previous unsuccessful surgical treatment.
Offer fertility preservation counseling to women at risk of cumulative ovarian damage, particularly before planned ovarian surgery and before the age of 35 years..	Do not repeat ovarian surgery as a strategy to improve fertility after prior operative failure.
Apply prognostic tools such as the Endometriosis Fertility Index as supportive aids within a broader individualized clinical framework.	Do not underestimate the cumulative impact of endometriosis and surgical interventions on long-term ovarian function.
Ensure shared decision-making, clearly aligning therapeutic strategies with patient preferences and time-sensitive reproductive priorities.	Do not pursue maximal intervention when proportionate, timely treatment better preserves reproductive potential and outcomes.

Overtreatment, particularly in the form of excessive or poorly indicated surgical intervention, represents a major threat to future fertility. Ovarian surgery, especially excision of endometriomas, is consistently associated with a significant and sometimes irreversible reduction in ovarian reserve, reflected by declines in AMH levels, antral follicle count, and oocyte yield in assisted reproductive technologies (ART).^(25,27)^ Beyond the direct loss of functional ovarian tissue, surgery may induce ischemic injury, vascular compromise, and postoperative adhesions, further impairing reproductive potential and increasing surgical morbidity.^(25,27)^ For this reason, major societies, including the American Society for Reproductive Medicine, emphasize that expectant management or direct referral to ART is often preferable in asymptomatic women or in those with small endometriomas, particularly when ovarian reserve is already compromised.^(3,27)^

Conversely, delayed treatment—especially postponement of ART in women with advancing age, prolonged infertility, diminished ovarian reserve, or advanced-stage disease—carries substantial reproductive risk. Endometriosis is a progressive condition, and its negative impact on fertility is compounded by age-related declines in oocyte quantity and quality.^(4,15)^ In these settings, prolonged expectant management or repeated low-yield interventions may result in missed opportunities for pregnancy and reduced cumulative live birth rates.^(15,25)^ The literature consistently supports early initiation of ART in women with advanced disease, low ovarian reserve, or failed prior surgery, as treatment delays may further compromise the likelihood of success.^(15,25)^

In addition to reproductive consequences, delayed or ineffective treatment may allow disease progression, leading to worsening pain, increased anatomical distortion, and greater surgical complexity if intervention is ultimately required.^(4,27)^ Thus, both extremes, unnecessary surgery and unjustified delay, can adversely affect fertility outcomes.

Optimal management therefore depends on individualized, multidisciplinary decision-making that integrates disease severity, symptom burden, age, ovarian reserve, duration of infertility, prior treatments, and patient preferences. Surgery should be reserved for well-defined indications, such as pain refractory to medical management, organ dysfunction, suspicion of malignancy, or technical barriers to ART, while ART should be promptly initiated when prognostic factors are unfavorable.^(3,25,27)^ Early counseling regarding fertility preservation is essential for women at risk of cumulative ovarian damage.

The goal in endometriosis-associated infertility is not maximal intervention, but timely and proportionate treatment, balancing preservation of ovarian reserve with avoidance of unnecessary delay, in order to optimize reproductive outcomes and long-term quality of life.

## Final considerations

Endometriosis is a complex and heterogeneous condition with a well-established negative impact on female fertility. Its effects are mediated through multiple mechanisms, including pelvic inflammation, anatomical distortion, impaired ovarian reserve, compromised oocyte quality, altered endometrial receptivity, and, in some cases, the consequences of surgical treatment. As a result, the evaluation and management of infertility in women with endometriosis must be individualized, evidence-based, and aligned with reproductive goals, disease phenotype, symptom burden, and patient preferences. Over recent years, growing evidence has expanded the understanding of endometriosis beyond its role in infertility alone. Endometriosis, particularly in its severe forms and when associated with adenomyosis, has been increasingly linked to adverse pregnancy and neonatal outcomes, including miscarriage, preterm birth, hypertensive disorders of pregnancy, placenta-related complications, and low birth weight. These findings reinforce the concept that endometriosis may influence reproductive health across the entire reproductive continuum, from conception to pregnancy outcomes. Clinical decision-making should not focus exclusively on fertility outcomes, but rather consider the broader reproductive context. Women with endometriosis should receive comprehensive counseling that addresses not only the chances of conception, whether spontaneous or assisted, but also the potential implications for pregnancy, while avoiding unnecessary medicalization. Importantly, pregnancy in women with endometriosis should not be universally classified as high risk; however, individualized obstetric follow-up may be appropriate in selected cases, particularly in those with deep endometriosis, adenomyosis, severe disease phenotypes, or additional maternal risk factors. In this context, the management of endometriosis-associated infertility and pregnancy requires close collaboration between gynecologists, reproductive specialists, and obstetric care providers. An integrated, patient-centered approach, grounded in current evidence and shared decision-making, offers the best opportunity to optimize reproductive outcomes, ensure safe pregnancies, and improve overall reproductive health in women living with endometriosis.

## National Commission Specialized in Endometriosis of the Brazilian Federation of Gynecology and Obstetrics Associations (Febrasgo)

President:

Ricardo De Almeida Quintairos

Vice-president:

Marcia Mendonça Carneiro

Secretary:

Carlos Augusto Pires Costa Lino

Members:

Marcia Cristina Franca Ferreira
Raquel Papandreus Dibi
Julio Cesar Rosa e Silva
Helizabeth Salomão Abdalla Ayroza Ribeiro
Carlos Alberto Petta
Eduardo Schor
João Nogueira Neto
João Sabino Lahorgue Da Cunha Filho
Marco Aurelio Pinho De Oliveira
Marcos Tcherniakovsky
Mauricio Simoes Abrao
Sidney Pearce Furtado
Sergio Podgaec
Omero Benedicto Poli Neto

## National Commission Specialized in Assisted Reproduction of the Brazilian Federation of Gynecology and Obstetrics Associations (Febrasgo)

President:

Rivia Mara Lamaita

Vice-president:

Rui Alberto Ferriani

Secretary:

Pedro Augusto Araujo Monteleone

Members:

Maria Do Carmo Borges De Souza
Rafaella Gehm Petracco
Alexandre Vieira Santos Moraes
Karina De Sá Adami Goncalves Brandao
Claudio Barros Leal Ribeiro
Eduardo Pandolfi Passos
João Antonio Dias Junior
Marta Curado Carvalho Franco Finotti
Natalia Ivet Zavattiero Tierno
Paula Andrea De Albuquerque Salles Navarro
Paulo Gallo De Sá
